# A Central Clearing Clinic to Provide Mental Health Services for Refugees in Germany

**DOI:** 10.3389/fpubh.2021.635474

**Published:** 2021-02-01

**Authors:** Malek Bajbouj, Patricia Panneck, Sibylle-Maria Winter, Carlos Ajami, Jihad Alabdullah, Max Benedikt Burger, Anja Haberlandner, Eric Hahn, Andreas Heinz, Isabella Heuser, Armin Hoyer, Ulrike Kluge, Marion Aichberger, Dimitris Repantis, Stefanie Schreiter, Joachim Seybold, Igor Sutej

**Affiliations:** ^1^Department of Psychiatry, Campus Benjamin Franklin, Charité – Universitätsmedizin Berlin, Berlin, Germany; ^2^Department of Psychiatry, Campus Mitte, Charité – Universitätsmedizin Berlin, Berlin, Germany; ^3^Department of Child and Adolescent Psychiatry, Campus Virchow Klinikum, Charité – Universitätsmedizin Berlin, Berlin, Germany; ^4^Medical Directorate, Charité – Universitätsmedizin Berlin, Campus Mitte, Berlin, Germany

**Keywords:** refugees, mental health, posttraumatic stress disorder, affective disorder, stress, migration health

## Abstract

**Objective:** To determine migration related distress pattern in refugees and feasibility of a *de novo* established, central low-threshold outpatient clinic serving more than 80,000 newly arrived refugees in the metropole of Berlin.

**Methods:** In an observational cohort study the relative prevalence of major psychiatric disorders by age, place of living within berlin, language and region of origin were assessed in a refugee cohort from 63 nationalities speaking 36 languages.

**Findings:** Within 18 months, a total of 3,096 cases with a mean age of 29.7 years (11.7) have been referred from all 12 districts and 165 of 182 subdistricts of Berlin to the CCC. 33.7% of the patients were female. The three most frequent diagnoses were unipolar depression (40.4%), posttraumatic stress disorder (24.3%), and adjustment disorder (19.6%).

**Conclusion:** The present data gives insight into the distribution of mental disorders in a large sample of refugees and provides evidence that a CCC is an effective service to quickly and broadly provide psychiatric consultations and thus to overcome classical barriers refugees usually experience in the host communities. In Berlin, Germany, and Europe treatment resources for this population should focus on stress and trauma related disorders.

## Introduction

As a consequence of armed conflicts in embattled countries, more than sixty million people worldwide had been forced to leave their home countries according to recent UNHCR assessments (1). Although most forced migrants were seeking shelter in the countries directly neighboring the war regions, Central Europe has increasingly become one of the main destinations of larger transnational migration streams (2). As a consequence, since summer 2015 more than one million refugees have found their way to and stayed in Germany (3).

A large proportion of those refugees have witnessed the cruelties of civil war and experienced exceedingly stressful journeys to Central Europe. In addition, the process of resettlement in a novel environment with unfamiliar habits, norms and expectations, restrictive policies in regard to residence status, limited access to main stream health services, the lack of other basic infrastructures and the living conditions in provisional shelters all increase insecurity and uncertainty among the newcomers, often leading to ethnic discrimination and social exclusion (4). This in sum leads to an accumulation of emotional distress presumably resulting in increased prevalence rates of mental disorders within the group of refugees as compared to the general population (5–8). While the exact response pattern to stress and trauma experiences in this specific population after more than 2 years still remain unclear, it is beyond all questions that the overall demand in mental health services surmounted the capacities available within the existing German health care structures (9, 10).

Given the large number of potentially affected individuals, mental health stakeholders across Germany fiercely debate how the newcomers could best be provided access to a health care system, in which services of mental health care often are impeded by societal, individual, and structural barriers. Such barriers comprise the lack of knowledge about symptoms of mental disorders and treatment possibilities within the health care system and the still existing and widespread stigmatization of mental disorders. In addition to those general barriers, refugees experience further difficulties in form of language barriers and often culturally engrained different disease and treatment models (10–13).

Taken together, Germany faced a situation, in which a largely unprepared mental health care system needed to provide culture and trauma sensitive diagnostic and therapeutic procedures for a large number of refugees. As the health system was unable to bear this challenge without additional capacities, new mental health care models were needed to provide fast and low-threshold access to mental health care in order to diagnose, prioritize and treat refugees with mental disorders.

To address this need, we established in Berlin Germany's first central clearing clinic (CCC), an institution, in which refugees regardless of their legal and insurance status are seen at short notice. We here report results of the first six quarters of the CCC, in which the largest so far cohort of refugees (*n* = 3.096) in Germany underwent mental health screening.

## Methods

### Study Population and Outreach Activities

In the time frame between February 10th 2016 and July 28th 2017 there had been a total of 4.635 contacts which have been aggregated to a total of 3.549 cases. From these a total of 453 cases have been excluded from further analysis because patients did not show up to complete the diagnostic procedure or the diagnosis remained unclear after two contacts.

All assessments were performed in the central clearing clinic, which is centrally located and situated in an area well-known to refugees since it hosted the registration authority for all newly arrived refugees in Berlin until May 2016.

All refugee housing facilities were informed about the availability of psychiatric services at the CCC on the day before opening (February 9th 2016). Additional outreach activities for refugee registration authorities, social workers, teachers, psychologists and volunteers working in the housing facilities were offered every 4 to 8 weeks providing information about the CCC and basic knowledge in culture-sensitive diagnosis and treatment of trauma and stress related symptoms.

This observational study was performed in accordance with International Conference on Harmonization Good Clinical Practice guidelines and the principles of the Declaration of Helsinki. The Charité ethics committee approved the protocol of this retrospective study.

### Appointment Procedure and Psychiatric Assessment

Appointments were made via telephone and through email by social workers, volunteers, physicians, or by refugees themselves. A nurse with experience in mental health care made an initial estimate on case severity in order to adjust waiting time and give preference to the more severe cases to optimize resource allocation. There were also slots for immediate emergency contacts, for example for suicidal patients, after acute deterioration of symptoms or when the continuity of medication was crucial.

All psychiatric assessments were conducted by physicians experienced in transcultural psychiatry. The team of physicians consisted of two psychiatrists for adults, both working full time and one doctor for children and youth psychiatry who was working only part time at the CCC during 2016, but whose attendance was increased to a fulltime presence in 2017. Daily presence of at least one psychiatrist who spoke the most frequent language (Arabic) as a native language was ensured. Average assessment time was 1 h for adults and 90 min for children and adolescents. Some patients only had one single appointment. These cases either did not need further attendance or they were directly transferred to other institutions, such as outpatient clinics of psychiatric hospitals in Berlin. In other cases, follow up appointments at the CCC were scheduled to complete diagnostics or to offer further psychiatric support when referral could not be organized in a timely manner or for other reasons, lack of translators being the most common one.

At the CCC a range of the most commonly used psychiatric drugs was available on site. When physicians saw an indication to initiate pharmacological treatment, medication could directly be provided to the patients. Additionally, psychotherapeutic short-term interventions were offered as a group program to Farsi and Arabic speaking women.

### Translation Techniques

Language barriers was addressed in three ways: (i) a native Arabic speaking physicians provided care for the bigger part of Arabic speaking patients; (ii) interpreters for the main languages (Arabic and Farsi/Dari) were present in the CCC all the time, and (iii) for other languages on-demand interpreters and/or a video-based interpreter service was in use. These video-based consultations were conducted in front of a screen which connected a permanently available professional interpreter to psychiatrist and patient via audio and video. Although this medium certainly influenced the interaction the system was overall well-accepted by psychiatrists and patients.

### Analysis

We described the diagnoses of refugees who have been referred to the CCC in the mentioned time frame. We reported distribution of clinical syndromes and disorders involved, by age and sex. Data were analyzed on SPSS 21.

## Results

### Sociodemographic Data

**Figure 1:**
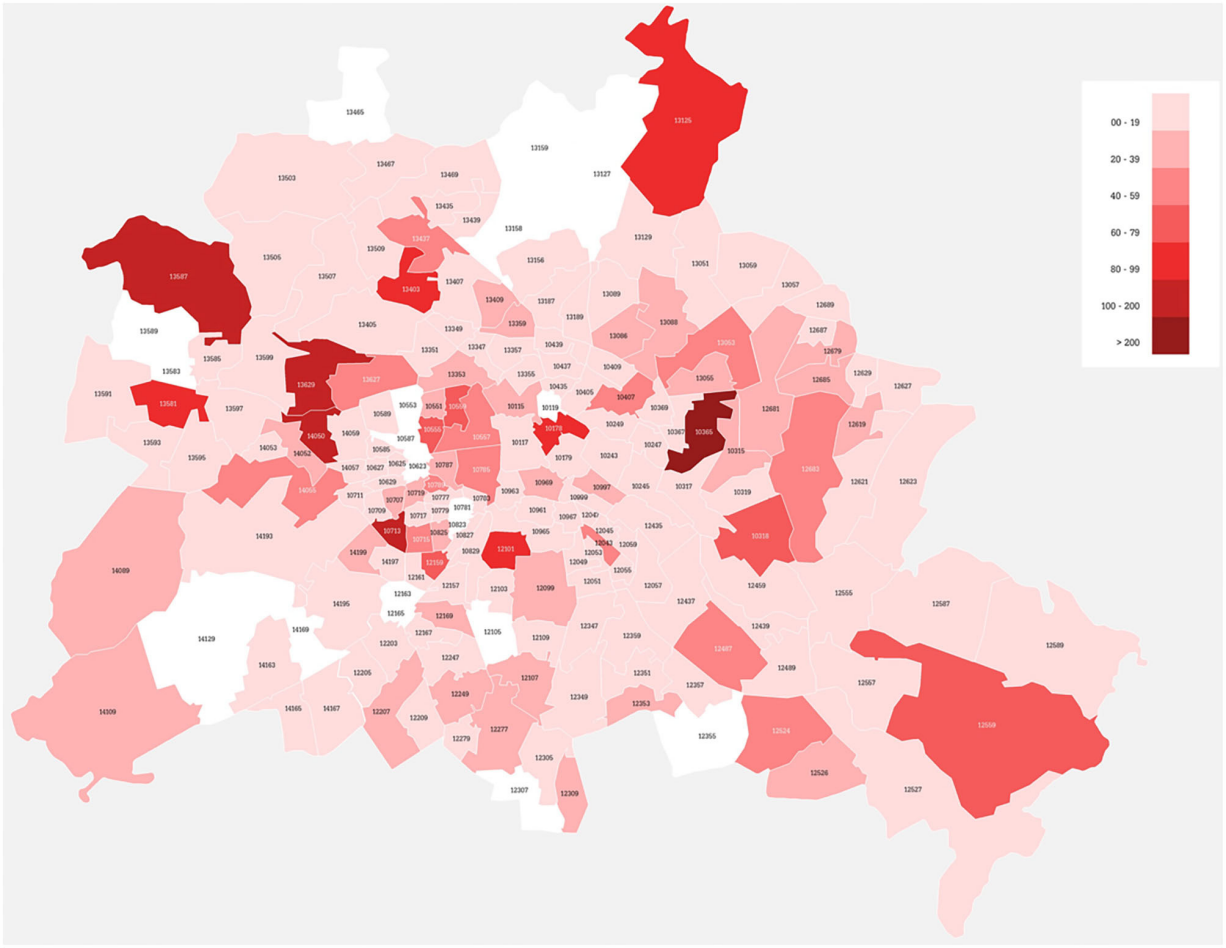
Sub-districts within Berlin using the mental health services of the central clearing clinic (CCC) after 72 weeks. Coloring indicate the number of patients that have been sent to the CCC with darker colors indicating a high number and lighter colors a low number of patients. Please note that the services have been utilized by patients coming from 165 out of 182 subdistricts.

**Figure 2:**
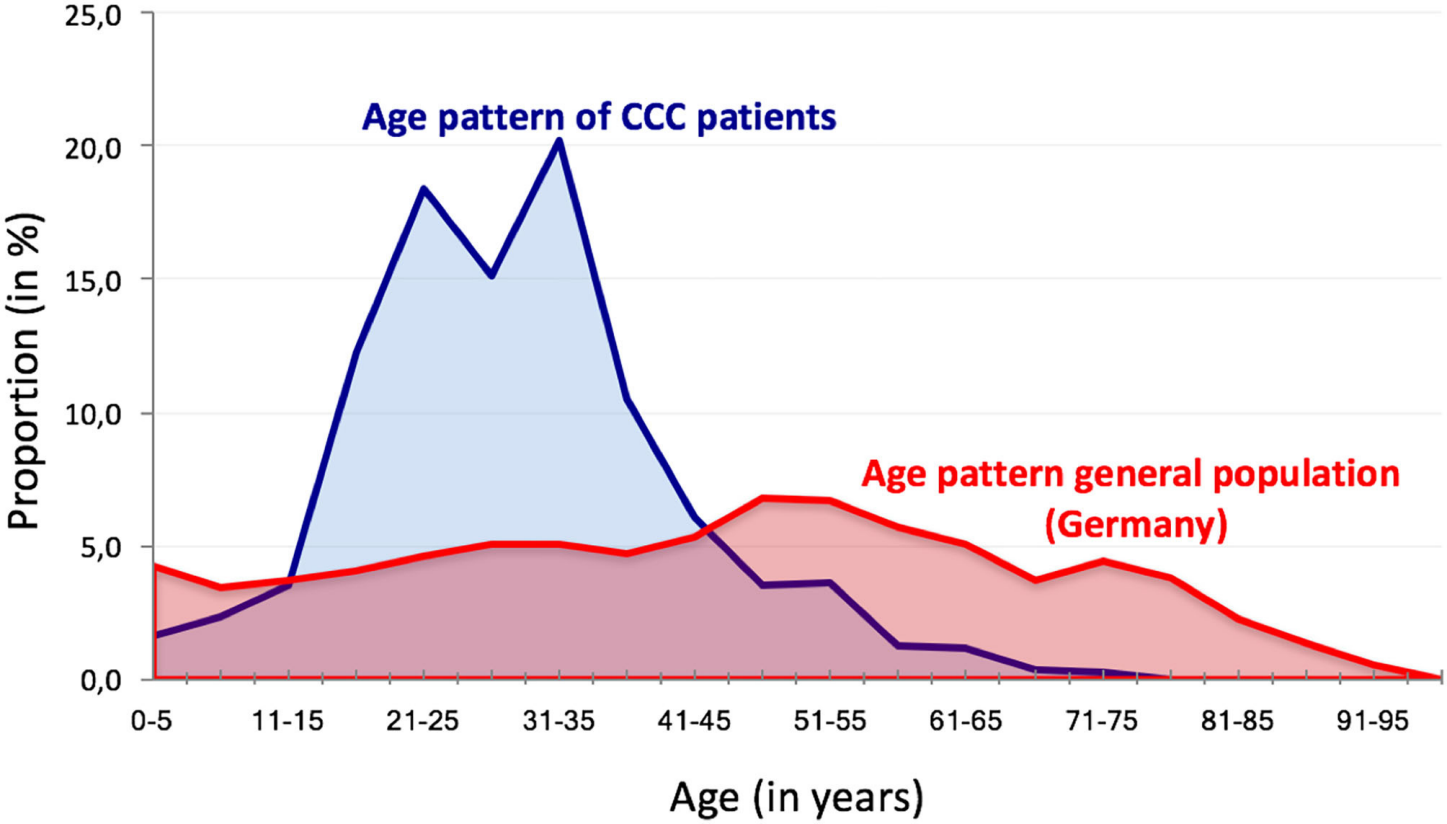
Age pattern of the CCC patients compared to the general population in Germany.

Over 72 weeks (from February 10th 2016 to July 28th 2017) the CCC had a total of 4.635 contacts with 3.096 refugees (see Supplementary Figure 1) from all twelve districts and 165 out of 182 sub-districts of Berlin (see Figure 1). The investigated population consisted out of 2.052 male and 1.044 female refugees with a mean age of 29.7 years (SD 11.7). The age pattern significantly differed from the pattern of the general German population with a clear peak and focus in the age range between twenty and 40 years (see Figure 2). Seven Hundred Sixty two contacts (16.4%) and 542 patients (17.5%) referred to the CCC were younger than 18 years (see Figure 2). Further characteristics of the investigated population are shown in Table 1.

**Table 1:** Number of contacts and cases and basic sociodemographic data of the studied population of refugees.

		**All**	**Male**	**Female**	**% Female**
All	Contacts	4.635	3160	1.475	31.8
	Cases	3.096	2.052	1.044	33.7
	Mean age (SD)	29.7 (11.7)	27.9 (11.2)	32.4 (12.3)	–
Adults	Contacts	3.871	2.589	1.282	33.1
	Cases	2.554	1.638	916	35.8
	Mean age (SD)	32.6 (12.0)	31.0 (11.3)	35.0 (12.3)	–
Children and	Contacts	764	571	193	25.3
adolescents	Cases	542	414	128	23.6
	Mean age (SD)	13.1 (13.3)	13.7 (12.1)	11.2 (14.0)	–

### Clinical Data

The most frequent disorders were unipolar depression (40.4%), posttraumatic stress disorder (24.3%), and adjustment disorder (19.6%). Notably, in 7.4% of all patients referred to the CCC patients did not show any clinical syndrome which could have been classified according to DSM5 or ICD 10. Unipolar depression was more frequent in female refugees whereas addiction and psychotic syndromes were diagnosed more often in male refugees (further details in Table 2).

**Table 2:** Total number of diagnoses in male and female refugees referred to the CCC between February 10th 2016 and July 27th 2017 (M, male; F, female).

		**Unipolar DP**	**PTSD**	**Adjustment disorder**	**No pathology**	**Anxiety disorder**	**Addiction**	**Psychotic disorder**	**Bipolar disorder**
All	*n*	1.249	753	607	229	142	140	137	24
	%	40.4	24.3	19.6	7.4	4.6	4.5	4.4	0.8
M	*n*	757	503	404	151	87	126	117	19
	%	36.9	24.5	19.7	7.4	4.2	6.1	5.7	0.9
F	*N*	492	249	203	78	55	14	20	5
	%	47.1	23.9	19.4	7.5	5.3	1.3	1.9	0.5
% F		39.3	33.1	33.4	34.1	38.7	10.0	14.6	20.8

### Discussion

Main results of the present investigation were (i) that a central clearing clinic is a feasible and probably superior institutional strategy to provide mental health care, and (ii) that stressful and traumatic life and flight experiences are associated with complex psychopathological reaction pattern with affective disorders, posttraumatic stress disorders and adjustment disorders being the most prominent disorders.

As a consequence of the steep rise of transnational migration Germany is becoming progressively ethno-culturally diverse, posing challenges for the countries' population and economy as well as for the refugees. This includes issues pertaining to the social and cultural inclusion of people into a receiving society, to social equality, education, labor market, democratic participation, social cohesion, and to the health care system. At the same time, the influx of people from ethnically and culturally heterogeneous backgrounds might spark progress toward an inclusive society benefiting from the variety of languages, cultural and ethnic diversity and the values and norms connected to it. More specifically, the challenges within healthcare comprise the availability of transculturally trained experts, techniques to overcome the language barrier and as a consequence the question whether the mental health care services should be provided in a centralized vs. a decentralized fashion.

### Characteristics of the Central Clearing Point

Typical Western European mental health care institutions are usually not experienced in working with refugees from heterogeneous countries and cultures, who often present unfamiliar and diverse histories of mental disorders and traumata. Especially diagnostic evaluation of psychiatric disorders is associated with particular difficulties: different concepts of illness and mental health (13), varying expressions of psychological distress as well as a lack of acceptance and trust in an unknown health care system (13). Potential consequences are misdiagnoses, which might lead to delayed adequate treatment with significant emotional distress for the patient and their relatives as well as an additional financial burden to the health care system.

Additionally, the absence of professional interpreters is often an essential obstacle. Recent studies reveal that patients who face language barriers receive unfavorable medical care (14, 15). In order to prevent such negative consequences, the use of professional interpreters is highly needed (16–19). But often financial funding of interpreters is unclear and health care institutions have to face the additional high financial burden (20). The availability of professional interpreters, respective bilingual physicians was a major advantage of the CCC. The financial as well as the organizational burden could not have been carried out by the regular mental health care system. We saw patients of 36 different languages (Supplementary Table 2) with Arabic and Farsi being by far the most common languages.

By conceptualizing the CCC as a central access point located next to the central registration authority for refugees, we alleviated the access to mental health care. This centralized set up of the CCC counteracts the pre-existing organization of mental health care facilities in Berlin, which traditionally aim to provide supply in every district of the city. Those community based mental health services have the advantages of (i) being close to the homes of the patients and such being more accessible, (ii) facilitating the collaboration between psychiatrists/psychologists and social workers, institutions etc. of a respective district, (iii) setting clear responsibilities regarding the mental health care in Berlin, (iv) and enabling the inclusion in the regular health care system instead of developing parallel structures which may enhance barriers and exclusion on the long run.

A central clearing point contrasts this model, but have been broadly utilized from the refugee population (as indicated in Figure 1). A reason might be that the available general mental health services did not have the resources to provide sufficient care within an appropriate time span: the group of refugees was even after arrival in Berlin a highly mobile group. Often refugees had to move several times within Berlin from provisionary refugee camps to permanent housings. For many refugees, the CCC became a stable contact they could return to whilst having to make an odyssey through different accommodations and institutions during the first months in Germany.

#### Priorisation Strategies in Mental Health

Especially in those countries which have become the primary destination of migration of populations from civil war regions, the comparatively high trauma and stress load dares for novel solutions in the field of mental health addressing the need for a quick and substantial response and at the same time acknowledging the composition, threshold and extend of available resources within the traditional system.

Such solutions can be inspired from concepts of humanitarian aid (9) or emergency medicine, that usually address situations, in which a quick response is required in an environment with limited resources. In such settings, in which treatment resources are insufficient to treat all patients immediately, a priorisation system (“triage”) is an effective approach to allot therapies efficiently. The concept, first described by Dominique Jean Larrey (19) during the Napoleonic Wars is nowadays a standard framework for many emergency medical services and a tool often used in mass-casualty incidents, e.g., in disaster medicine. Triage in this context refers to distinguishing between different levels of patients' needs and referring them to adequate treatment options, it does not exclude any patient from required treatment but rather helps to provide targeted interventions. Psychosocial and disaster behavioral health issues in situations affecting a large number of patients are used in broader concepts such as the continuous integrated care. However, triage concepts that exclusively address mental health services which are entrusted with decisions regarding the allocation of resource-intense therapies or in-patient treatments have rarely been implemented. The main considerations hindering such concepts is at first the general approach that every patient should be provided with the needed treatment as soon as possible regardless of the severity of the disorder. Second, unlike in emergency medicine, treatment decisions in most mental disorders happen rather on long-term considerations and often without an immediate life-threatening consequence making the implementation of a triage system more complex.

In the CCC we were able to proof that a priorisation system can be applied in mental health care. Advantages of the CCC were that with relatively little means it was possible to provide fast mental health care services to a larger population otherwise hard to access. This was indicated by the fact that patients were referred from all districts of Berlin. Even though they received an evaluation within a relatively short time span, the waiting time for appointments rather increased over the months which supports the high need for a mental health care contact point. While those patients that needed psychiatric care urgently could be identified faster and transferred to appropriate institutions for further treatment, less severe cases could also be identified and partly supported in the CCC, such unburdening the more specialized institutions and making more efficient use of the available resources such as specialized trauma therapies. By that, it was avoided that limited capacities were used ineffectively and immoderately. The needs for costly interventions were evaluated through psychiatrist with experience in intercultural work, ensuring that the indication was checked professionally.

Shortcomings of the CCC were that for those not in need of specialized treatments low-threshold interventions such as stress relief groups or support by social workers were not (yet) broadly available. To overcome this issue, we offered short term interventions in a group setting, which however were only available to a small group of our patients.

### Pattern of Trauma and Stress Response in Refugees

So far, there is little evidence on the prevalence of mental disorders among refugees arriving in Germany in the last 3 years (20). The data from the CCC are corresponding to some extend with existing international data, showing an increased risk for depression and post-traumatic stress disorders in refugee populations compared to the general population (21, 22). In general, the rates of almost all psychiatric disorders diagnosed at the CCC show increased rates compared to the general German population (DEGS Study) (23). While comparing our findings to prevalence dates of the general German population one must take into account that our data relies on a preselected younger subpopulation as compared to the general population (see Figure 2). In consequence, disorders more prevalent in the population below 40 years are likely to be overrepresented. Of importance: as only cases that presented noticeable psychiatric problems were referred to the CCC, one must assume lower prevalence rates in the general refugee population. On the other hand, the data from the 3.096 included cases allow useful insights into the complexity of psychiatric disorders with a broad variety of clinical syndromes and suggest the need for further differentiated studies on the impact of refugee status and refugee living conditions as well as flight circumstances and discrimination experiences on the mental health status of the refugee population in Germany. Of importance, the finding that affective disorder, PTSD and adjustment disorder are the leading diagnoses support the current and future need for an increase in psychosocial and psychotherapeutic offers for refugees.

### Limitations

However, we need to point out some difficulties and potential blurs of the preliminary data presented here: (i) diagnostic evaluation in the CCC was mainly based on clinical interviews. In order to respond to the huge demand and to keep the screening procedure feasible, we refrained from a systematic use of psychologic questionnaires, which were not available in all languages. (ii) In addition, reliability of our diagnostic evaluation therefore depended crucially on the quality of interpretation and may differ between e.g., Arabic native speaking psychiatrists and consultations conducted with interpreters. (iii) In the procedure of application for asylum, medical attestations become a certain form of informal currency that may influence chances of success. A lot of patients consulting in the CCC asked for medical attestations, in some cases this demands seemed to be the main reason for consultation. It was sometimes difficult to estimate how and to what extent this demand influenced the description of symptoms relevant for the diagnostic assessment. (iv) Moreover, the structure of the CCC leads to the fact that mainly the severe cases were referred and thus data are not representative of the refugee population, albeit the large sample size.

In conclusion, we were able to demonstrate that the concept of a central institution prioritizing mental health needs of individuals with a high stress and trauma load is feasible and –importantly- well-accepted. The CCC concept might be scalable and serve as a model for other settings where populations with a high stress load are coming into receiving countries with limited resources within the mental health care system. It may not only improve mental health of refugees but may also serve as an intervention against the frequently reported perception and experience of discrimination, that may further hamper the adaptation process for newcomers during resettlement (24, 25). To avoid exclusive health care structures, the current challenge is to integrate emergency services including translators into general mental health care and its local organization including service sectors and local networks of hospitals and outpatient services.

## Data Availability Statement

The raw data supporting the conclusions of this article will be made available by the authors, without undue reservation.

## Ethics Statement

The studies involving human participants were reviewed and approved by Charité ethics committee. The patients/participants provided their written informed consent to participate in this study.

## Author Contributions

MB, PP, IS, JA, and JS analyzed the data and drafted the report. PP, CA, MBB, AHa, AHo, SS, and DR assisted in clinical case confirmation. AHe, S-MW, IH, MB, JS, EH, UK, and MA contributed to the methodology and study design. JS, IH, and AHe edited the drafted report. All authors contributed to the article and approved the submitted version.

## Conflict of Interest

The authors declare that the research was conducted in the absence of any commercial or financial relationships that could be construed as a potential conflict of interest.
