# Supplementary material for: A Central Clearing Clinic to Provide Mental Health Services for Refugees in Germany
Source: Front Public Health. 2021 Feb 1;9:635474. doi: 10.3389/fpubh.2021.635474 (PMC7901997; doi:10.3389/fpubh.2021.635474)
Supplement: Supplementary file 1 [file Table_1.DOCX]

S1: Number of patients (in blue) and contacts (blue plus orange) in the first six quarters (each lasting 12 weeks) between February 10^th^ 2016 and July 27^th^ 2017 (total numbers)

S1: List of all 63 nationalities and number of contacts in the first six quarters from February 10^th^ 2016

| Afghanistan | 1550 | Kuwait | 3 |
| --- | --- | --- | --- |
| Syria | 1449 | Nigeria | 3 |
| Iraq | 731 | Togo | 3 |
| Iran | 238 | Chad | 3 |
| Lebanon | 166 | Ukraine | 3 |
| Albania | 95 | Uzbekistan | 3 |
| Eritrea | 92 | India | 2 |
| Egypt | 80 | Jordan | 2 |
| Pakistan | 69 | Cameron | 2 |
| Palestine | 50 | Kirgizstan | 1 |
| Somalia | 48 | Kurdistan | 2 |
| Turkey | 39 | Saudi-Arabia | 2 |
| Serbia | 29 | Azerbaijan | 1 |
| Bosnia | 28 | Bulgaria | 1 |
| Russia | 23 | Burkina Faso | 1 |
| Guinea | 21 | Cuba | 1 |
| Chechen | 20 | Ivory Coast | 1 |
| Morocco | 18 | Georgia | 1 |
| Moldavia | 18 | Kazakhstan | 1 |
| Gambia | 17 | Kenia | 1 |
| Libya | 16 | Croatia | 1 |
| Tunisia | 13 | Mali | 1 |
| Turkmenistan | 13 | Mauritania | 1 |
| Armenia | 8 | Macedonia | 1 |
| Benin | 8 | Rumania | 1 |
| Algeria | 6 | Senegal | 1 |
| Tadzhikistan | 6 | Sierra Leone | 1 |
| Bangladesh | 5 | Spain | 1 |
| Yemen | 4 | Sudan | 1 |
| Sahara | 4 | Vietnam | 1 |
| Ghana | 3 |  |  |

S2: List of all 36 languages and number of contacts in the CCC from February 10^th^ 2016 and July 28^th^ 2017

| Arabic | 2.393 |
| --- | --- |
| Farsi | 1.606 |
| English | 122 |
| Dari | 118 |
| Albanian | 88 |
| Kurdish | 86 |
| Russian | 77 |
| Tigrinya | 74 |
| French | 55 |
| Kurmanji | 49 |
| Urdu | 44 |
| German | 43 |
| Somali | 42 |
| Sorani | 41 |
| Pashtun | 39 |
| Serbian | 24 |
| Bosnian | 22 |
| Turkish | 13 |
| Bengalian | 5 |
| Dendi | 5 |
| Armenian | 4 |
| Fulani | 3 |
| Romani | 3 |
| Chechen | 3 |
| Swahili | 2 |
| Amharian | 1 |
| Gorani | 1 |
| Italian | 1 |
| Croatian | 1 |
| Madrinka | 1 |
| Panjabi | 1 |
| Spanish | 1 |
| Turkmenian | 1 |
| Usbek | 1 |
| Vietnamese | 1 |
| Yoruba | 1 |

S4: Number of patients in the first six quarters (each lasting 12 weeks)
